# Creation of a Patient Information Video to Improve the Patient Experience of the Surgical Assessment Unit

**DOI:** 10.7759/cureus.57926

**Published:** 2024-04-09

**Authors:** Heather Pringle, Pranu Ragatha, Aye Myat Myintmo, Hazel Awarah, Waihim Chung, Rob Bethune

**Affiliations:** 1 General Surgery, Torbay and South Devon NHS Foundation Trust, Exeter, GBR; 2 Surgery, Royal Devon University Healthcare NHS Foundation Trust, Exeter, GBR; 3 General Practice, Sherwood Forest Hospitals NHS Foundation Trust, Sutton-in-Ashfield, GBR; 4 General Practice, Nottingham University Hospitals NHS Foundation Trust, Nottingham, GBR; 5 Colorectal Surgery, Royal Devon University Healthcare NHS Foundation Trust, Exeter, GBR; 6 Colorectal Surgery, Royal Devon and Exeter Hospital, Exeter, GBR

**Keywords:** emergency general surgery, video feedback, emergency medical service, acute care surgery and trauma, quality improvement projects

## Abstract

Background: Patients who attend emergency surgical services are entering an unfamiliar environment whilst often being unwell and in pain. Patient satisfaction in emergency surgical units is often low due to poor communication with attendees and long wait times.

Methods: A pilot patient questionnaire identified areas where patient satisfaction was low during attendance at the surgical assessment unit (SAU). The aim of this intervention was to improve patient satisfaction with their experience whilst attending the SAU. An education video was filmed to address the areas where services were falling short of expectations, and this was played in the waiting room. Further questionnaire results tailored the frequency of the video to achieve maximum impact.

Results: Data were collected at three time points: firstly, prior to the introduction of the video (n=34); secondly, with the video played hourly (n=15); and finally with the video played every 30 minutes at a higher volume (n=15). Mean satisfaction scores after the final cycle improved to 7.3 from 4.9 (p=0.0009). Additionally, 94% of patients agreed that the video was in keeping with their personal experience of the SAU and agreed that the video improved their understanding of what to expect from the visit.

Conclusions: Interventions that improve communication with patients and adjust their expectations play an important role in improving patient satisfaction and their overall perception of care. This can be achieved with a simple patient information video.

## Introduction

Patients attending the surgical assessment unit (SAU) are acutely unwell and often in pain. This is a stressful time for patients who are not familiar with acute secondary care units and are apprehensive about pending investigations and possible need for surgery. This is compounded by further uncertainties including long waiting times and busy staff with other priorities, resulting in patients feeling like they are being ignored. Healthcare professionals can forget how stressful a visit to a hospital can be for patients as we are so accustomed to the day-to-day life of working in the health service.

Patient-reported satisfaction is an important goal in emergency services as it reflects the quality of care that the patient receives both on an individual scale and departmentally [1]. Even when the patients have received technically good quality care, the overall care perceived by the patient is poor in the context of poor satisfaction [2]. Satisfied patients take on more information from the medical team and as a consequence are more likely to follow medical advice, resulting in less frequent visits to the hospital [3].

What is the SAU?

In this university teaching hospital, the SAU is the entry point for emergency surgical patients. The nursing team carries out simple bedside investigations before review by a member of a surgical team. The patients are assessed by the corresponding on-call surgical team (general surgery, urology, vascular surgery, plastic surgery, ENT, orthopaedics, or gynaecology), appropriate investigations ordered, and treatment commenced. A small number of patients at a low risk of deterioration are asked to return the following day for further investigations and clinical review through an established “home and back” pathway. This means that the patients will occasionally spend more than one day in the waiting room pending review.

The problem

Pilot work performed using patient questionnaires explored levels of patient satisfaction and potential areas where care was falling short of expectations. A key theme derived from this work was a lack of sufficient explanation of what the patients could expect while at the SAU. Expectancy theory in psychology suggests that satisfaction is based on the difference between expectations and what is delivered [1,4]. For the purposes of this article, patient satisfaction can be defined as when the patient’s own expectations of care are met or surpassed [5]. Following this principle, early management of patient expectations through clear and transparent communication may improve patient satisfaction regardless of any improvements in clinical care delivered.

The key criticisms from patients were a lack of transparency around the length of waiting times, unclear reasons for prolonged waiting times to see the medical team, and a lack of explanation of routine processes prior to medical review (for example, the need for blood tests, urine samples, and duplicated nursing screening questions).

Aim

The aim of this project was to improve patient satisfaction with their experience whilst attending the SAU by use of a patient information video. The video that was created was educational to explain the pressures on the SAU whilst providing patients with clear instructions on what to expect from their visit.

This article was previously presented as an abstract oral presentation at the UK-Iceland Trainee Collaborative 2nd Annual Conference on the 10th of June 2022.

## Materials and methods

We used the Model of Improvement with Plan, Do, Study, Act (PDSA) cycles to help design our interventions and enact change based on the findings. Baseline data were gathered in a pilot questionnaire to record patient experience and patient perceptions on how their experience could be improved. Using the results from this, a patient information video was filmed, and the same questionnaire was repeated. There was one patient information video filmed with no change in the video content on subsequent questionnaire results. The video frequency and volume were altered after patient feedback.

Measures

The primary outcome measure was the self-reported patient satisfaction score (1-10), where a score of 10 represented perceived excellent care. The questionnaire collected further information regarding their stay on a 5-point Likert scale. In terms of the analysis, a score of 5 reflected “strongly agree.” There was a free text section for patients to leave written feedback on their experience and suggested areas for improvement.

The questionnaires were voluntary, and all data collected was anonymised. Questionnaires were made available in the admission packs along with a leaflet explaining the study. After completion, the patient placed the questionnaire in a box on the ward to be collected by the study team in the evening. An example of the patient questionnaire can be seen in Table 1.

**Table 1:** Sample section of the patient questionnaire

Please tick the relevant box for the following statements:	Strongly agree	Agree	Neutral	Disagree	Strongly disagree
I understand why I am attending the surgical assessment unit.					
The staff were welcoming and approachable.					
I didn’t have to wait long for all my procedures to be performed.					
The procedures went smoothly.					
I prefer to be seen and have all my tests/scans done on the same day rather than across 2 days, even if it means spending longer in the hospital.					
I understand what my test results mean.					
Overall, I am satisfied with the services I have been provided.					

Analysis

The outcome data were quantitative and are presented as a mean. Given small sample sizes, an assumption of normal distribution could not be made, so a student’s T-test was used to test for statistical significance.

Ethical considerations

Patient feedback should never affect the care that they receive; therefore, to ensure that patients feel comfortable and safe providing feedback, care was taken to preserve their anonymity.

Pilot data collection

There was anecdotal evidence that patients were not finding the care they received in SAU acceptable (for example, patients self-discharging prior to review or putting pressure on the nurses to find a doctor).

The original information gathered included wait time, timing of investigations, and time of senior review. With the results of the pilot study, the decision was made to make a business case for a patient information video. A script was written by a committee of doctors on the general surgical team with approval from the hospital communications department. The concepts covered were designed to address the key issues highlighted by the results of the patient questionnaires.

Creation of the video and feedback

The patient information video was a two-minute 40-second brief of what patients can expect during their visit to the SAU. The content was structured in an interview format featuring staff from the SAU. The topics included in the video were the role of the SAU, a description of the patient pathway, a description of the composition and responsibilities of the on-call surgical team, and reasons for potential delays. There was also an explanation of the potential need for the patient to come back the following day for further investigations.

The video was played on a television monitor in the SAU waiting room. The video was also put onto the hospital intranet site where patients had the opportunity to view the video prior to arrival.

## Results

Data were collected at three time points: prior to the introduction of the video (n=34); video played hourly (n=15); and video played every 30 minutes at a higher volume (n=15). Table 2 shows the mean results of the main questions.

**Table 2:** Results of the Likert scale section of the patient questionnaire

Relevant Questionnaire Section	Pilot Data	After Video	Video More Often
Patient understands reason for attendance	3.6	3.5	3.6
Staff welcoming and approachable	3.6	3.6	3.5
Patient felt they did not have to wait long for tests	2.3	2.2	2.5
Prefer to be sorted in one day	2.9	3.1	3.0
Patient understands what results mean	3.2	3.5	3.3
Overall satisfaction (out of 10)	4.9	7.1	7.3

Not all patients saw the video whilst they were waiting. Compared to the video playing every hour, the video playing more frequently at a higher volume increased uptake by patients (40% during the second cycle had seen the video whilst waiting compared to 80% in the second cycle). A higher proportion also agreed/strongly agreed when asked if they could clearly hear and understand the content of the video (67% in the second cycle vs 100% in the third cycle).

Overall, the mean satisfaction scores following the introduction of the video playing every 30 minutes, at a higher volume, were significantly improved compared to baseline (mean 7.3 vs 4.9, p=0.0009, two-tailed t-test assuming unequal variance).

**Figure 1:**
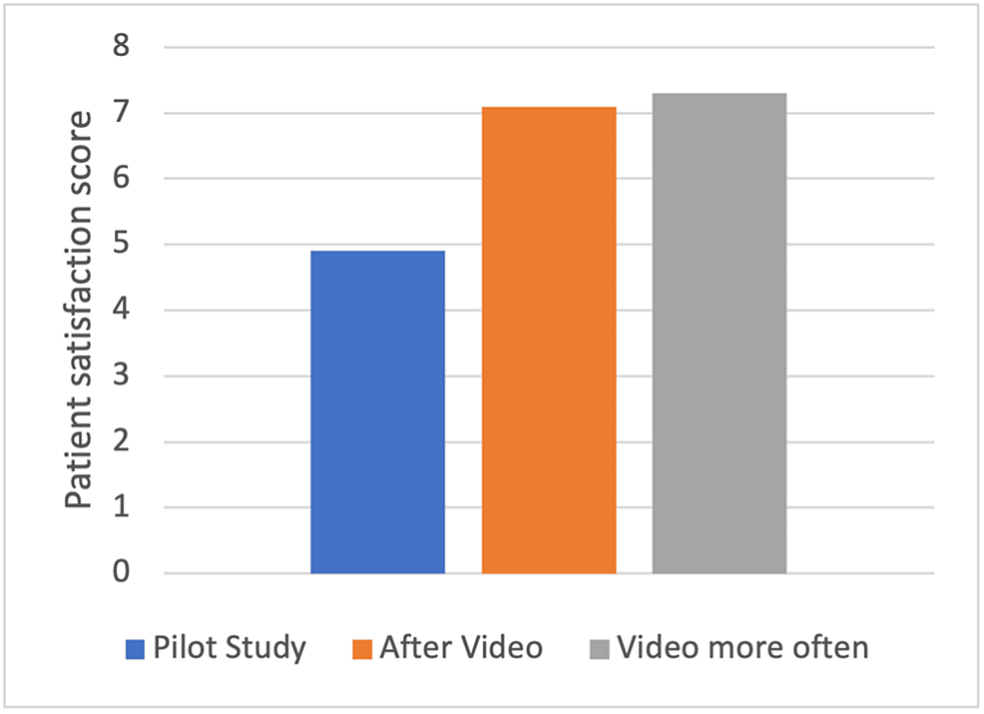
Bar chart of patient satisfaction after each data collection

Patients provided opinions on the video, and 94% of patients agreed that the video content was in keeping with their own personal experience at the SAU. Similarly, 94% of patients agreed that the video improved their understanding of what to expect from the visit. Figure 1 shows a graph of the results.

## Discussion

This project improved patient satisfaction by introducing a patient information video addressing patient-focused limitations of their visit to the SAU. It has been previously described that healthcare expectations have at least two elements. Idealistic expectations are what patients expect a healthcare system to be capable of delivering and predictive expectations are more realistic, based on previous experience of healthcare [5]. This project informed the latter to define the standard of care patients can expect to receive.

When the pilot data were being collected, it was noted that the average waiting time for patients on the SAU was eight hours. The original aim of this project was to reduce waiting times, which has been shown to improve patient satisfaction [6,7]. Nurse-led discharges in ambulatory patients have improved patient experience and are more cost-effective in other trusts [8]. However, with the staffing level, this could not be done safely in this department. The realisation that reducing the wait would be impossible given that funding and staffing constraints literature showed that patients could be "willing to wait" based on proactive communication with patients and improved perceived value of the service. Following this, the idea of the patient information video evolved [9].

Complaints in healthcare often stem from a lack of communication [10]. In an ideal world, effective communication between staff and patients would not require the aid of a pre-recorded video. However, the reality of providing care in busy acute units is that we often fall short of giving patients the time they deserve. This video is a small stepping stone towards addressing this concern. The benefit of a patient information video is that it does not impact on the day-to-day running of the medical team who often have to prioritise emergencies.

Patient satisfaction and patient perception of the care they have received is a requisite of quality assurance, and both clinicians and managers must take an interest in being accountable for the services provided [5,11,12]. Public satisfaction in the National Health Service has been declining with the lowest level since 1997 recorded in 2024 at 24% [13]. It is essential that we take on board what patients want from their care in order to set the steps in motion to restore their faith in the NHS [13-15].

The front-line staff in this project were motivated and determined to improve patient experience. The healthcare assistants were on board and drew patients' attention to the questionnaire. A number of staff volunteered to be in the video. There was minimal training of staff required to engage them in the improvement process. This all stemmed from the determination for improvement and the project team spending time with the staff explaining the objectives. This is transferrable to any area of quality improvement.

Limitations

As common with quality improvement projects, a key limitation is its applicability to different settings. The intervention was developed based on specific patient feedback for the SAU. Given the multiple dynamic factors involved in delivering care on the SAU, it can be difficult to account for all of these. The questionnaires were handed out to patients who were motivated to fill it in, and therefore not all patient interactions with the service were recorded. A longer period of data collection after each intervention, with a larger sample size, would provide a more reliable picture. A business case allowed funding for the video. The crew was professional but costly. This may not be possible in other trusts where there is not an acknowledged impact on patient satisfaction. On top of this, if there is a change to the geography of the unit or the standard operating procedures, the video would become immediately out of date.

## Conclusions

Patients who are referred for emergency surgical review are often in pain and in an unfamiliar environment. Patients have an expectation of what awaits them and may not understand the pressures on departments. In order to ensure expectations meet reality, information needs to be given to patients at the right time. The introduction of a patient information video to patients in the waiting room successfully informed patient expectations, which in turn resulted in improved patient satisfaction. In the current, turbulent climate of the NHS, a simple intervention of a patient information video has been shown to play a role in improving patient satisfaction, and articles like this can set in motion discussions on how we can restore public faith in our health service.
